# *Palmul*-Tang, a Korean Medicine, Promotes Bone Formation via BMP-2 Pathway in Osteoporosis

**DOI:** 10.3389/fphar.2021.643482

**Published:** 2021-03-26

**Authors:** La Yoon Choi, Mi Hye Kim, Yeon Kyung Nam, Ju Hee Kim, Hea-Young Cho, Woong Mo Yang

**Affiliations:** ^1^ Department of Convergence Korean Medical Science, College of Korean Medicine, Kyung Hee University, Seoul, South Korea; ^2^ College of Pharmacy, CHA University, Seongnam, South Korea

**Keywords:** osteoporosis, traditional herbal medicine, palmul-tang, bone integrity, BMP-2

## Abstract

Osteoporosis is a common skeletal disease in post-menopausal women. *Palmul*-tang, an herbal medicine, has been treated for gynecological disease such as anemia, anorexia, anti-fatigue, unspecified menstruation and female infertility in East Asia. In this study, ameliorative effects of *Palmul*-tang soft extracts (PMT), a Korean Medicine, on osteoporosis were investigated. Ovariectomized (OVX) osteoporotic ICR mice were intragastrically administrated PMT for 4 weeks. The level of bone mineral density (BMD) was analyzed in bone tissues by dual X-ray absorptiometry. The bone medullary cavity and deposition of collagen were investigated by histological analysis. In addition, the BMP-2 signaling-related molecules, osteoblastic differentiation and formation markers, were determined in femoral tissues. The levels of BMD and bone mineral content were significantly increased in tibia, femurs and LV by treatment of PMT. PMT replenished bone marrow cavity and increased collagen deposition in bone marrow cells of femur. In addition, administration of PMT recovered serum ALP, bALP, osteocalcin and calcium levels in osteoporotic mice. Moreover, PMT treatment up-regulated the expressions of BMP-2, RUNX2 and OSX with its downstream factors, ALP, OPN and BSP-1, in the femoral tissues. Taken together, PMT restored the bone minerals and improvement of bone integrity by bone-forming BMP-2 signaling pathway. These results demonstrate that PMT could be an ameliorative agent for osteoporosis.

## Introduction

Osteoporosis is a skeletal disease by occurring the impairment of osteoblast and osteoclast balances (Chen et al., 2018). During the procession of osteoporosis, the reduction of bone mass and deterioration of bone tissue leads to augmentation of bone fragility, thereby lowering bone mineral density (BMD) and increasing the risk of bone fractures such as wrist, hip and vertebrae (Kanis, 2002; Cheng et al., 2011). More than 30% of women over the age of 50 estrogen deficient which also causes environmental changes such as increased treatment costs and poor quality of life (Dickenson et al., 1981). Accordingly, management and prevention of osteoporosis is the most representative public health issue for the assignment of global strategies (Lewis et al., 2019).

A common prescription for treatment of osteoporosis is hormone therapy, bisphosphonates and supplements such as calcium and vitamin D (Bowring and Francis, 2011). The mode of action by anti-osteoporosis drugs is associated with improvement of balances for abnormal function of osteoblast and osteoclast that increase the bone mass and offset the bone loss (Tanaka et al., 2005; Nanjundaiah et al., 2013). However, the possibility of experiencing side effects including hot flush, breast cancer, blood clots, heart failure and osteonecrosis of jaw still remains an obstacle to be overcome and drives us to find new prospective drugs for improving osteoporosis (Nam et al., 2012; Tella and Gallagher, 2014).

Traditional herbal medicine in Korea is used to treat various diseases because of its low side effects and high efficacy in prevention and treatment (Yuan et al., 2016). *Palmul-*tang, also known as *Paljin*-tang in Korean, *Hachimotsu-to* in Japanese and *Bawu*-tang in Chinese, is a herbal formula which is consists of eight herbs, *Panax ginseng* C. A. Meyer, *Atractylodes macrocephala* Koidz, *Poria cocos* F. A. Wolf, *Glycyrrhiza uralensis* Fischer, *Rehmannia glutinosa* (Gaertner) Liboschitz, *Paeonia lactiflora* Pallas, *Ligusticum officinale* (Makino) Kitag and *Angelica gigas* Nakai (Eom et al., 2017; Jin et al., 2017). *Palmul-*tang has traditionally been prescribed for anemia, anorexia, anti-fatigue, and nerualgia (Heo et al., 2003; Park et al., 2004a; Park et al., 2004b; Ma et al., 2007b; Shin and Shin, 2012; Kim and Bak, 2015). Especially, *Palmul-*tang has been used for women diseases such as unspecified menstruation, reproductive activities and female infertility (Ma et al., 2007a). Also, *Palmul*-tang was used for women diseases such as unspecified menstruation, reproductive activities and female infertility (Ma et al., 2007a). Recent study showed inhibitory effects of *Palmul*-tang on inflammatory mediator production related to NF-κB, MAPK and HO-1 pathway in macrophages (Oh et al., 2014). According to the traditional theory including Korean medicine, traditional Chinese medicine and Kampo medicine, *Palmul*-tang has been known for improving the basis of metabolism and enhancing the organ activity by nourishing “qi” and enriching “blood.” Tonifying “qi” induces bone marrow to nourish the bone, resulting in growth of skeleton (Wang et al., 2016b). Interruption of “blood” circulation is highly involved in increasing osteoclastic activities leading to induction of cumulative bone loss as a crucial factor for bone growth (Fricke and Krokowski, 1975; Alagiakrishnan et al., 2003). In addition, *Palmul*-tang is a combination of *Sagunja*-tang and *Samul*-tang, which is reported to be effective for osteoporosis (Lee et al., 1995; Shim et al., 2011; Yim et al., 2018). Based on the recent studies, we anticipated that *Palmul*-tang soft extracts (PMT) has ameliorative effects on post-menopausal osteoporosis. In the present study, the effects of PMT on bone loss was investigated by assessing BMC and BMD levels and bone-forming BMP-2 signaling pathway-related molecules were determined in bone tissues.

## Materials and Methods

### Samples

PMT (Lot. #8001), which is a standardized Korean medicine for health insurance, was obtained from Kyoungbang pharmacy Inc. (Incheon, Korea). The total volume of PMT is 10 g per pack and composed of *Panax ginseng* C. A. Meyer, *Atractylodes macrocephala* Koidz, *Poria cocos* F. A. Wolf, *Glycyrrhiza uralensis* Fischer, *Rehmannia glutinosa* (Gaertner) Liboschitz, *Paeonia lactiflora* Pallas, *Ligusticum officinale* (Makino) Kitag and *Angelica gigas* Nakai (Table 1). Voucher specimen PMT (Lot. #8001) is deposited in our laboratory.

**TABLE 1:** Components of *Palmul*-tang soft extract one pack.

Y	Family	Source	Weight (mg)
*Panax ginseng* C. A. Meyer	Araliaceae	Korea	420
*Atractylodes macrocephala* Koidz	Asteraceae	Korea	495
*Poria cocos* F. A. Wolf	Polyporaceae	Korea	225
*Glycyrrhiza uralensis* Fischer	Fabaceae	Korea	660
*Rehmannia glutinosa* (Gaertner) Liboschitz	Orobanchaceae	Korea	720
*Paeonia lactiflora* Pallas	Paeoniaceae	Korea	495
*Ligusticum officinale* (Makino) Kitag	Apiaceae	Korea	645
*Angelica gigas* Nakai	Apiaceae	Korea	495

### High-Performance Liquid Chromatography

Three packets (45 g) of PMT was thoroughly mixed, extracted and filtered to make a test liquid for injecting into the Alliance HPLC e2695 system with 2489 UV/Vis detector. A full scan spectrum in positive ESI mode was obtained for the main ingredient identification of PMT. Then the reference standards of atractylenolide I and atractylenolide III were accurately weighed and dissolved in methanol. The chromatographic separations of the analytes were conducted on Kromasil C18 column (4.6 × 159 mm, 3.5 μm). The mobile phase consisted of (A) water and (B) acetonitrile (30:70, v/v) flowed at a rate of 1.0 ml/min. Then 10 μL of the sample was injected for the analysis. Contents of atractylenolide I and atractylenolide III were 0.033 ± 0.0010 mg and 0.039 ± 0.0002 mg, respectively (n = 3). (Supplementary Figure S1).

### Animals

All experiments were conducted with the approval of the Committee on Care and Use of Laboratory Animals of Kyung Hee University, Seoul, Korea (KHSASP-19-097). Female ICR mice aging of 5 weeks old were obtained from RaonBio Inc. (Yongin, Korea). All mice were acclimated at least 1 week under standard housing conditions (22–24°C, 12 h/12 h light/dark cycle) and had freely access food and water. The experimental groups divided into 6 groups (n = 7); 1) sham (blank control), 2) OVX (negative control), E2 (OVX + 17β-estradiol, 10 μg/kg, positive control), PMT 61.7 (OVX + PMT 61.7 mg/kg), PMT 617 (OVX + PMT 617 mg/kg), PMT 6170 (OVX + PMT 6170 mg/kg).

### Experimental Protocol

All mice were intraperitoneally injected with avertin (Sigma-Aldrich, St. Louis, United States) and shaved dorsal midline skin. The shaved skins were longitudinally incised and operated sham or removed bilateral ovaries. Exposed skin and muscle were closed with silk 4-0 suture (AILEE co., Busan, Korea) and applied povidone iodine on surgical area to disinfect the skin. Following induce osteoporosis for 10 weeks, Day 70, OVX-operated groups were assigned to 5 groups in accordance with body weights. The PMT samples were administrated 5 days per week for 4 weeks; sham and OVX groups were orally treated with vehicle, E2 group was intraperitoneally injected with 10 μg/kg of 17β-estradiol, PMT groups were orally treated with dose of 61.7, 617, 6,170 mg/kg of PMT diluted in distilled water. The doses of PMT were selected from formula for dose translation by human equivalent dose (Nair and Jacob, 2016). The dosage of samples in use for human is 30 g/60 kg/day (3 packs). Dosage for human (30 g/60 kg/day) is converted into mice (6,170 mg/kg/day) by using human equivalent dose equation as high dosage of this study. During animal experiments, body weight was monitored by weekly. The body weights of osteoporotic mice showed almost same values in whole experimental periods without any adverse events. At the end of experiments, Day 96, the mice were sacrificed by cervical dislocation. The sacrifice mice dissected blood, tibiae, femora and L4∼L6 vertebrae (LV) to analyzed efficacy of PMT on osteoporosis.

### Dual Energy X-Ray Absorptiometry Test

To measure bone mineral content (BMC; g) and bone mineral density (BMD; g/cm^2^), excised tibiae, femora and LV were detected by using dual energy X-ray absorptiometry (DXA, Medikors, Seongnam, Korea).

### Histopathological Examination

Excised left femurs were fixed in 10% neutralized formalin for 24 h and demineralized using 0.1 M ethylene diamine tetra acetic acid (EDTA) for 2 weeks. After decalcification, femurs were dehydrated with a graded series of alcohol, xylene and paraffin. The femurs were embedded for sagittal sections in paraffin and harden in the freezer for 24 h. Paraffin blocks were sliced using microtome into 10 μm-thick and placed on gelatin-coated glass slides. To evaluate the recovery of bone marrow cavity and interstitial collagen, hematoxylin and eosin (H&E) staining and picrosirius red staining were performed. Stained sections were photographed with magnification ×400 under microscope.

### ELISA Analysis of Osteoblastic Factors

Collected blood sample were isolated by centrifuge at 17,000 rpm for 20 min and supernatants were kept at −20°C until use. The serum samples were moved at 4°C 1 day before usage. Serum alkaline phosphatase (ALP), bone-specific ALP (bALP) (AnaSpec, United States), osteocalcin (TaKaRa Bio Inc., Japan) and Ca (Nikken SEIL Co., Japan) levels were analyzed using enzyme-linked immunosorbent assay (ELISA) kit. The procedures were conducted according to manufacturer’s instructions.

### RT-PCR Analysis

To analyze osteoblastic markers, right femurs were pulverized using liquid nitrogen with mortar and pestle into fine powder. Powdered femurs were incubated with trizol (Invitrogen, United States) at 4°C for overnight and homogenized. Total RNA was extracted according to the manufacturer’s protocols. Total RNA was measured and synthesized cDNA using 1 μg RNA and Maxime RT premix (Invitrogen). Reverse transcription polymerase chain reaction (RT-PCR) was performed using Maxim PCR premix (Invitrogen). Osteoblastic markers specific primers were as follows: 5′-GCG​GTG​GAC​TGC​ACA​GGG​AC-3′ and 5′-CTA​CCC​TTC​CCC​GTG​GGG​GA-3′ for *bone morphogenetic protein 2* (BMP-2), 5′-CCG​CAC​GAC​AAC​CGC​ACC​AT-3′ and 5′-CGC​TCC​GGC​CCA​CAA​ATC​TC-3′ for *runt-related transcription factor 2* (RUNX2), 5′-TAA​TGG​GCT​CCT​TTC​ACC​TG-3′ and 5′-CAC​TGG​GCA​GAC​AGT​CAG​AA-3′ for *osterix* (OSX), 5′-TGG​AGC​TTC​AGA​AGC​TCA​ACA​CCA-3′ and 5′-ATC​TCG​TTG​TCT​GAG​TAC​CAG​TCC-3′ for *alkaline phosphatase* (ALP), 5′-CAT​TGC​CTC​CTC​CCT​CCC​GGT​G-3′ and 5′-GTC​ATC​ACC​TCG​GCC​GTT​GGG​G-3′ for *osteopontin* (OPN), 5′-GAG​CCA​GGA​CTG​CCG​AAA​GGA​A-3′ and 5′-CCG​TTG​TCT​CCT​CCG​CTG​CTG​C-3′ for *bone sialoprotein* (BSP-1), 5′-GGC​ATG​GAC​TGT​GGT​CAT​GA-3′ and 5′-TTC​ACC​ACC​ATG​GAG​AAG​GC-3′ for *glyceraldehyde 3-phosphate dehydrogenase* (GAPDH). The gene expressions were detected by 1% agarose gel and normalized to housekeeping gene, GAPDH. All bands were analyzed by image J software.

### Statistical Analysis

All data are analyzed by using GraphPad Prism 5 software (GraphPad Software Inc., La Jolla, CA, United States). Significance was determined by one-way analysis of variance (ANOVA) and Tukey multiple comparison tests. Data were presented as the mean ± standard error. In all analyses, *p* < 0.05 was taken to indicate statistical significance. All data underlying the study was deposited in our laboratory.

## Results

### Effects of PMT on BMC and BMD

**FIGURE 1:**
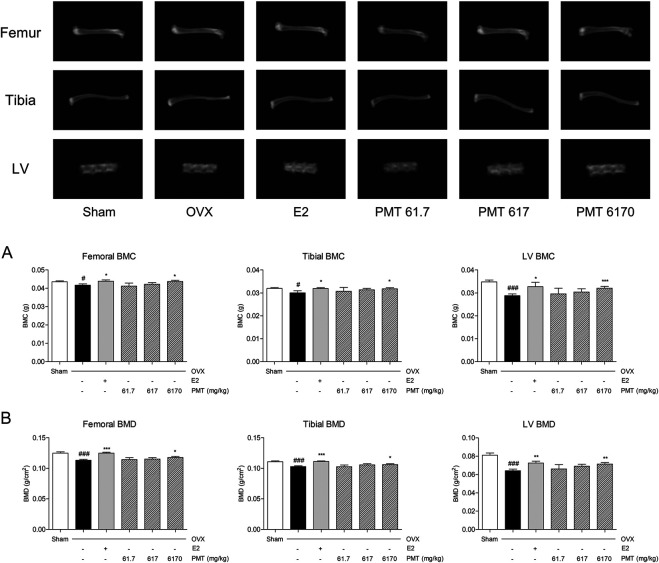
Effects of PMT on BMC and BMD in osteoporotic mice. Bone mineral content and bone mineral density of tibiae, femora and lumbar vertebrae were analyzed by DXA. **(A)** Levels of bone mineral content in tibia, femur and LV. **(B)** Levels of bone mineral density in tibia, femur and LV. Results are presented as mean ± standard error of the mean. ^#^ *p* < 0.05, ^##^ *p* < 0.01 and ^###^ *p* < 0.001 vs. Sham group; *p < 0.05, **p < 0.01 and ***p < 0.001 vs. OVX group. OVX, ovariectomized group; E2, 17β-estradiol group; PMT, *Palmul*-tang soft extracts; LV, lumbar vertebrae; BMC, bone mineral content; BMD, bone mineral density.

Femoral, tibial and LV were used to evaluate the levels of BMC and BMD. As shown in Figure 1A, levels of femoral, tibial and LV BMC was declined 4.33, 6.01 and 17.31% in OVX group compared to sham group and recovered 5.20, 6.33 and 13.65% in E2 group compared to OVX group. Treatment of PMT up-regulated BMC in femur, tibia and LV compared to OVX group, especially increased 4.69, 5.86 and 11.32% in 6,170 mg/kg of PMT. As similar as result from Figure 1B, levels of BMD on femora, tibial and LV were decreased 9.29, 7.12 and 20.87% in OVX group by PMT. Also, treatment of E2 increased levels of BMD by 10.24% in femora, 8.06% in tibiae and 13.10% in LV. In addition, 6,170 mg/kg of PMT improved levels of BMD, 3.97, 3.10 and 11.34%, respectively.

### Effects of PMT on Histological Changes

**FIGURE 2:**
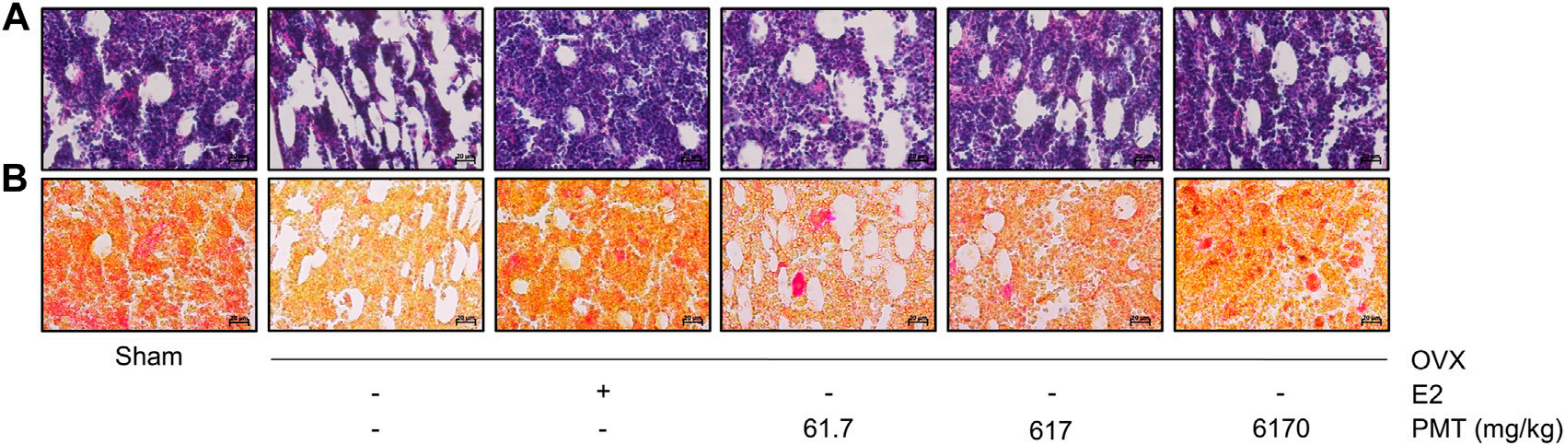
Effects of PMT on histological changes in femur. Evaluation of histological changes using hematoxylin and eosin staining for bone marrow cavity and picrosirius red staining for detecting deposition of collagen. Magnification of images were ×400. OVX, ovariectomized group; E2, 17β-estradiol group; PMT, *Palmul*-tang soft extracts.

The bone marrow cavity in femoral shaft was used to determine by H&E staining. In Figure 2A, OVX group appeared high adiposity of bone marrow compared to sham group, whereas treatment of E2 and PMT ameliorated bone marrow adiposity, particularly treated with 6,170 mg/kg of PMT. Picrosirius red staining showed quantitatively improvement of collagen fibers in femur. As demonstrated in Figure 2B, the collagen fibers were markedly aggravated in OVX group compared to sham group. However, treatment of E2 and PMT recovered expression of collagen fibers, especially 6,170 mg/kg of PMT treatment.

### Effects of PMT on Bone Specific Markers in Serum

**FIGURE 3:**
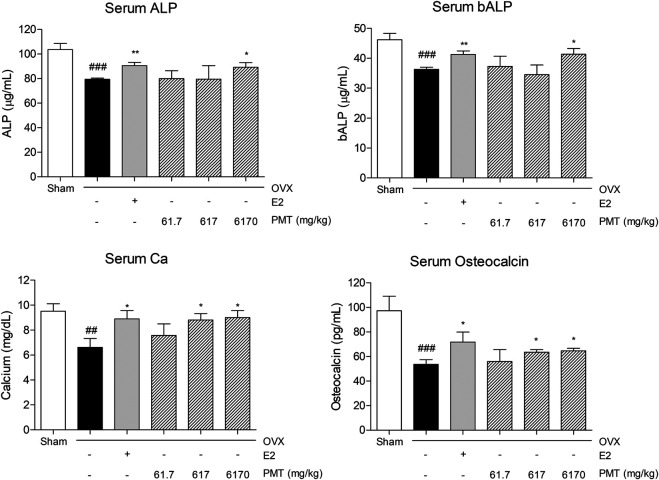
Effects of PMT on bone specific markers in serum. Secretion of bone specific markers in serum was analyzed by ELISA. Results are presented as the mean ± standard error. ^##^ *p* < 0.01 and ^###^ *p* < 0.001 vs. Sham group; *p < 0.05 and **p < 0.01 vs. OVX group. OVX, ovariectomized group; E2, 17β-estradiol group; PMT, *Palmul*-tang soft extracts; ALP, alkaline phosphatase; bALP, bone-specific alkaline phosphatase.

The ALP and bALP levels were decreased in OVX group, 23.29 and 21.43%, respectively, whereas E2 treatment increased levels of ALP and bALP compared to OVX group, 13.89 and 13.59%, respectively. In addition, the levels of ALP and bALP were recovered by administrating PMT compared to OVX group. Particularly 6,170 mg/kg of PMT treatment significantly increased expression levels by 12.31% in ALP and 13.86% in bALP. In addition, serum Ca levels were diminished 30.39% in OVX group, and rise 34.31% in E2 group. Treating PMT remarkably improved levels of Ca 32.96% in PMT 617 group and 36.04% in PMT 6170 group. Serum levels of osteocalcin, similar to Ca, was decreased 44.93% in OVX group and increased 34.07% in E2 group. Treatment with 617 and 6,170 mg/kg of PMT significantly advanced 18.7 and 20.85% Ca levels, respectively, in OVX-induced osteoporotic mice (Figure 3).

### Effects of PMT on Osteoblastic Markers in Femur

**FIGURE 4:**
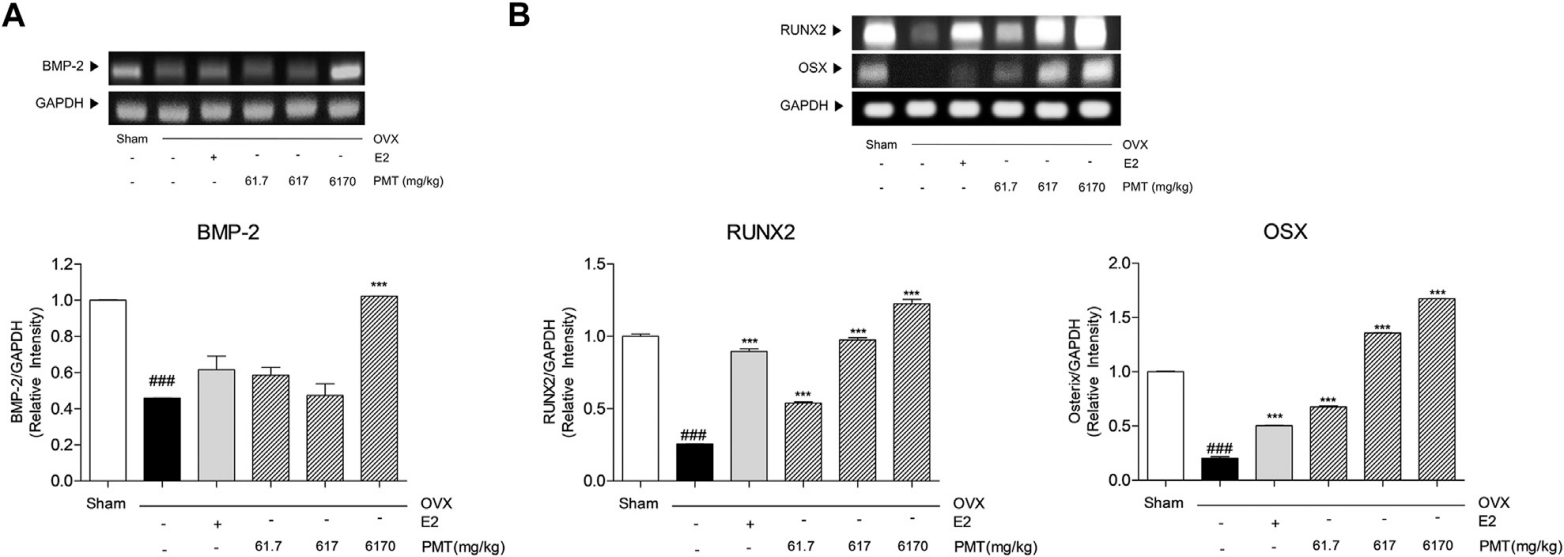
Effects of PMT on osteoblastic differentiation initiation markers in femur. Osteoblastic differentiation initiation markers were analyzed by RT-PCR. Results are presented as the mean ± standard error. ^###^ *p* < 0.001 vs. Sham group; ***p < 0.001 vs. OVX group. OVX, ovariectomized group; E2, 17β-estradiol group; PMT, *Palmul*-tang soft extracts; BMP-2, bone morphogenetic protein 2; RUNX2, Runt-related transcription factor 2; OSX, osterix.

**FIGURE 5:**
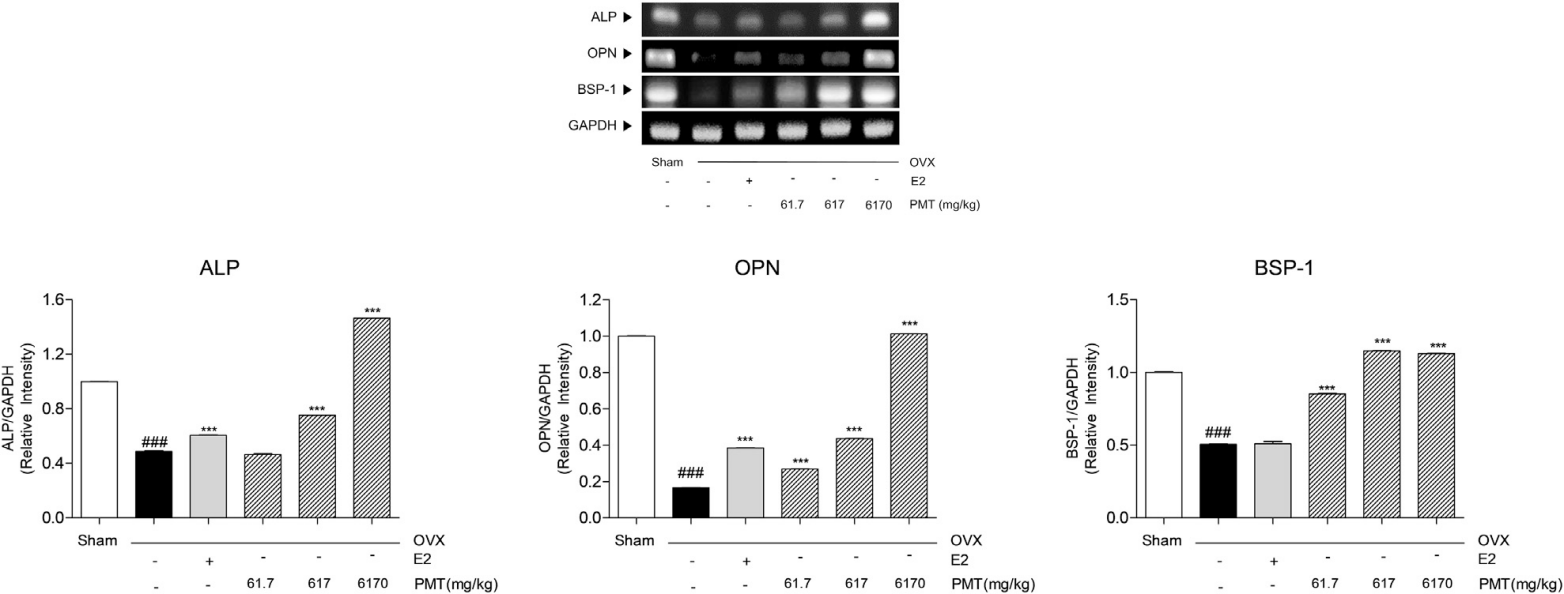
Effects of PMT on osteoblastic production markers in femur. Osteoblastic production markers were analyzed by RT-PCR. Results are presented as the mean ± standard error. ^###^ *p* < 0.001 vs. Sham group; ***p < 0.001 vs. OVX group. OVX, ovariectomized group; E2, 17β-estradiol group; PMT, *Palmul*-tang soft extracts; ALP, alkaline phosphatase; OPN, osteopontin; BSP-1, bone sialoprotein 1.

As shown in Figure 4A, BMP-2 was expressed in OVX group 54.14% lower than sham group, however, there was no significance in E2 group. While 6,170 mg/kg of PMT treatment recovered 122.63% higher than OVX group. Expression levels of BMP-2 in PMT 6170 group seems to be approximated with sham group. In addition, transcription factors in osteoblast, RUNX2 and OSX, were decreased 74.45 and 79.73%, respectively, in OVX group. Expression of RUNX2 and OSX were significantly increased 250.37 and 148.25%, respectively, treated with E2. The treatment three doses of PMT remarkably up-regulated expressions of RUNX2 and OSX dose-dependent manner, 1.1, 2.81 and 3.79 folds of RUNX2 and 2.34, 5.7 and 7.25 folds of OSX, respectively (Figure 4B). By regulating osteoblast differentiation, ALP, OPN and BSP-1 were lowly expressed in OVX group, 51.21, 83.33 and 49.41%, respectively. On the contrary, treatment of E2 highly expressed mRNA levels of ALP and OPN, 24.27 and 130.7%, respectively. However, there was no significance expression of BSP-1 in E2 group. Administration of PMT increased levels of ALP, OPN and BSP-1 dose-dependent manner. These markers were significantly expressed 60.95, 161.13 and 507.68% of OPN and 68.49, 127.12 and 123.36% of BSP-1, respectively. Additionally, expression levels of ALP were increased 54.27 and 200.06% by treating 617 and 6,170 mg/kg of PMT (Figure 5).

## Discussion

Postmenopausal osteoporosis is a metabolic and skeletal disease that has a high prevalence of age-related bone loss with porous and low levels of bone density (Garnero, 2014). BMD is commonly used in the clinics for the standards of osteoporosis, which is diagnosed with -2.5 or lower levels BMD T-score in the region of the femoral neck and lumbar spine (Kanis, 2002). In addition, the collapse of bone microstructure results in increases of the risk of fractures by reducing bone strength (Chesnut et al., 2001). Moreover, to form of mature osteoblast, collagen is essential extracellular matrix protein for increasing bone strength and remodeling (Elango et al., 2018). In this study, OVX-induced osteoporotic mice group, levels of BMC and BMD in femur, tibia and LV were lowered compared to sham-operated mice group. On the contrary, the levels of BMC and BMD were significantly improved in PMT-treated mice. In addition, PMT reduced bone marrow adiposity, which was promoted the deposition of collagen tissue in the bone marrow. Accordingly, PMT enhanced the levels of BMC, BMD and refilled a cavity with bone mineral which leads to improvement of bone integration.

Impaired osteoporotic bone is the imbalance of osteoblast and osteoclast (Aggarwal et al., 2012). Especially, postmenopausal osteoporosis reveals the inactivation of osteoblasts due to the insufficiency of estrogen, which disrupted the equilibrium of osteoblast and osteoclast (Paschalis et al., 2003). Promotion of osteoblastic activities, which lead to bone proliferation, maturation and formation, is recommended to prioritize for treating post-menopausal osteoporosis (Wang et al., 2016a). Bone turnover markers including ALP, bALP, Ca and osteocalcin, as specific and sensitive markers of bone formation, have been investigated in clinical and experimental studies in the early and late stages of osteoporosis (Risteli and Risteli, 1993). Several reports related to bone metabolism that the expression of ALP is maximized during bone matrix maturation (Alcantara et al., 2011). In addition, osteocalcin is a calcium-dependent biomarker that has a high affinity for the bone matrix to complete bone formation (Singh et al., 2015). In our study, levels of serum ALP and bone-specific ALP were lowly expressed in osteoporotic mice, while significantly recovered by administering PMT 6170 mg/kg. Additionally, OVX-induced mice treated with PMT augmented that diminished levels of serum Ca and osteocalcin. Thus, osteoblast differentiation is attributed to PMT by increasing levels of serum ALP, bALP, Ca and osteocalcin.

To clarify the underlying mechanism of PMT on the recovery of bone loss, osteoblastogenetic factors were investigated in femoral specimens. BMP-2 activates the expression of RUNX2 and OSX, which are mediated for osteoblast differentiation and new bone formation (Lee et al., 2003; Fan et al., 2013). In addition, bone-specific matrix proteins such as ALP, OPN and BSP-1 are also associated with osteoblast maturation for bone formation (Cowles et al., 1998). Hence, those genes are considered as the key markers of osteoporosis that newly construct a bone matrix. The present study determined that expression of osteoblastic markers BMP-2, RUNX2, OSX, ALP, OPN and BSP-1 were significantly decreased in OVX-induced osteoporosis while treating PMT increased those of osteoblastic markers in a dose-dependent manner. Therefore, PMT influences on osteoblasts to repair the balances of bone homeostasis and the formation of new bone matrix.

## Conclusion

Taken together, PMT has ameliorative effects on postmenopausal osteoporosis. In the previous study, *P. ginseng*, *P. lactiflora* and *L. officinale*, consists of *Palmul*-tang, is recently reported to enhance the osteoblast activities, leading to generation of new bone matrix (Yen et al., 2007; Kim et al., 2013; Dong et al., 2020). In addition, *A*. *macrocephala* and *P. cocos*, and decursin derived from *A. gigas* are known to ameliorate the development progress of osteoporosis by inhibiting osteoclast differentiation (Ha et al., 2013; Wang et al., 2016c; Hwang et al., 2020). Moreover, *G. uralensis* and *R. glutinosa* showed protective effects on osteoporosis by maintaining bone mineral density (Lim and Kim, 2013; Galanis et al., 2019). *Palmul*-tang, which is a combination of those eight herbs as a traditional herbal formula, was assumed to exhibit a synergic effect on bone formation in osteoporosis. In this study, PMT ameliorated bone loss in osteoporosis by activating osteoblastic markers such as BMP-2 signaling pathway, leading to improving bone integrity. The bone marrow adiposity cavity filled with collagen fibers to restore the bone marrow tightly. Moreover, the expressions of the BMP-2 signaling pathway were upregulated by PMT treatment, resulting in an increase of bone differentiation and formation. These results might provide evidence of expanding the medical category and the basis of medical insurance in Korea for postmenopausal osteoporosis.

## Data Availability

The original contributions presented in the study are included in the article/Supplementary Material, further inquiries can be directed to the corresponding author.
