# Supplementary figures and images for: Palmul-Tang, a Korean Medicine, Promotes Bone Formation via BMP-2 Pathway in Osteoporosis
Source: Front Pharmacol. 2021 Mar 26;12:643482. doi: 10.3389/fphar.2021.643482 (PMC8032944; doi:10.3389/fphar.2021.643482)

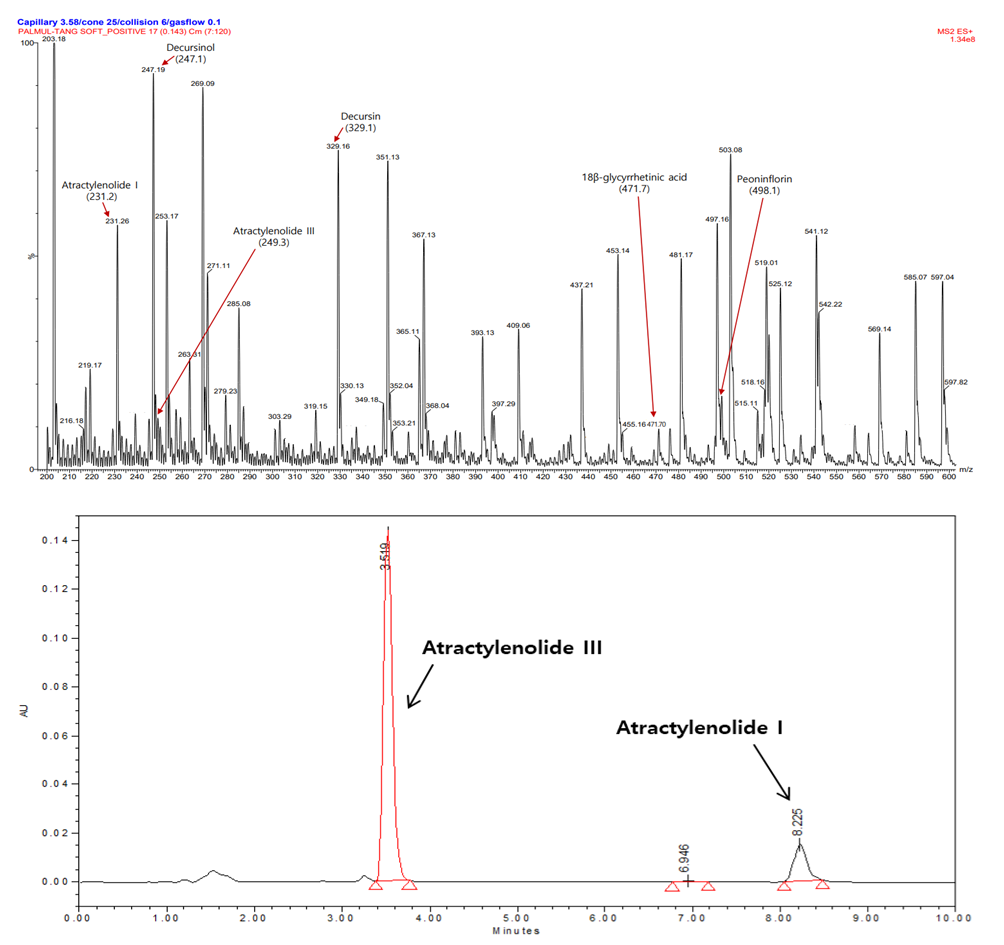

Supplement: Supplementary file 1 [file image1.tif]
